# Refractory Dry Eye Syndrome after Transconjunctival Excision of the Palpebral Lobe of the Lacrimal Gland

**DOI:** 10.3390/medicina57060608

**Published:** 2021-06-11

**Authors:** Yong-Jae Lee, Han-Sol Choi, Seong-Jun Park, Hae-Jung Sun, Sun-Young Jang

**Affiliations:** 1 Department of Ophthalmology, Soonchunhyang University Bucheon Hospital, Soonchunhyang University College of Medicine, 170, Jomaru-ro, Bucheon 14584, Korea; 124957@schmc.ac.kr (Y.-J.L.); 121966@schmc.ac.kr (H.-S.C.); apolo_kr2000@naver.com (S.-J.P.); 2 Department of Ophthalmology, Soonchunhyang University Seoul Hospital, Soonchunhyang University College of Medicine, 59, Daesagwan-ro, Yongsan-gu, Bucheon 04401, Korea; sunhj99@schmc.ac.kr

**Keywords:** dry eye syndromes, lacrimal apparatus, eyelids

## Abstract

The aim of the present study was to report two cases of refractory dry eye syndrome (DES) after transconjunctival excision of the palpebral lobe of the lacrimal gland. A 25-year-old female patient with a chief complaint of a palpable mass in both upper eyelids visited our medical center. Preoperative orbital computer tomography showed high-attenuation lesions in both lacrimal glands. Incisional biopsy of the lacrimal gland palpebral lobe via transconjunctival incision was performed in January 2019. At 1 month after the biopsy, a lack of tears and persistent corneal erosions were found in both eyes. Artificial tears, punctal occlusion, autologous serum eye drops, and therapeutic contact lenses were applied in an attempt to control the dry eye symptoms. The patient continues to suffer from intractable DES at 2.5 years after the procedure. The second case involved a 52-year-old female patient who visited our medical center with a chief complaint of a palpable mass in both upper eyelids. Bilateral orbital tumors were diagnosed with preoperative magnetic resonance imaging. An incisional biopsy of the lacrimal gland was performed. Immunoglobulin G4-related dacryoadenitis was confirmed through lacrimal palpebral lobe incisional biopsy. Intractable DES and corneal erosion of her left eye persisted thereafter. A transconjunctival incision is an effective approach for minimizing postoperative scars and is suitable for the biopsy of tumors that are visible through the conjunctiva. After a biopsy of the palpebral lobe of the main lacrimal glands, the secretion of reflex tears decreases due to damage to the secreting ducts of the main lacrimal glands. However, total tear secretion can be maintained by basal tear secretion from the accessory lacrimal glands. In this report, we describe two cases of refractory DES due to decreased total tear secretion, although only the palpebral lobes of the main lacrimal glands were biopsied.

## 1. Introduction

Dry eye disease (DED) is defined as a “multifactorial disease of the ocular surface characterized by a loss of homeostasis of the tear film” that is “accompanied by ocular symptoms in which tear film instability and hyperosmolarity, ocular surface inflammation and damage, and neurosensory abnormalities play etiological roles”, according to the International Dry Eye Workshop II [1]. Tear films are composed of mucin and aqueous and lipid layers. The mucin layer is derived from goblet cells of the conjunctiva, the aqueous layer from the lacrimal gland, and the lipid layer from the meibomian glands [2,3]. The aqueous component of tears is produced by the main lacrimal glands, accessory lacrimal glands, and corneal and conjunctival epithelia [4]. In 1966, Jones [5] stated that the main lacrimal gland is responsible only for reflex tearing, whereas the accessory glands of Kraus and Wolfring provide basal tear secretion; however, this description has been debated. The volume of tears secreted from these glands has not been determined. Moreover, previous reports describe contradictory results as to whether the accessory glands provide an adequate tear volume to prevent dry eye syndrome (DES) [6,7]. The main lacrimal gland is generally thought to be an indispensable source of the aqueous layer of tears; however, there is evidence that adequate tear secretion can exist in the absence of the main lacrimal gland [4].

Herein, we report two cases of refractory DES after transconjunctival excision of the palpebral lobe of the lacrimal gland, along with a literature review. This case report provides additional scientific information to elucidate the main lacrimal gland function in DES.

## 2. Case Reports

**Figure 1:**
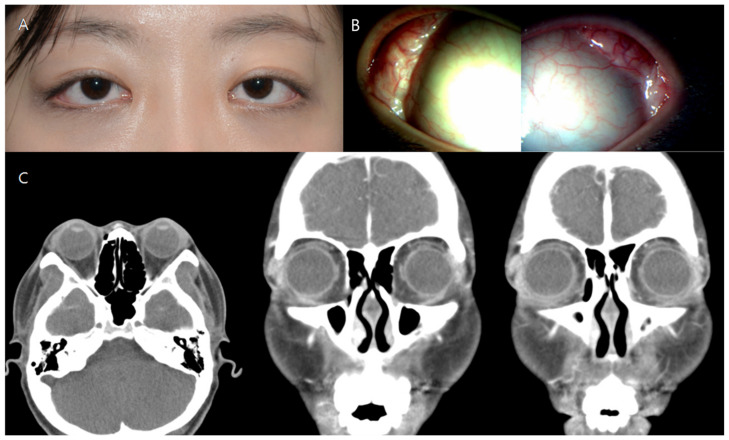
Preoperative images of Case 1. Both upper lids exhibited swelling (**A**) along with a protruding palpebral lacrimal lobe (**B**). Computed tomography showed a heterogeneous contrast-enhanced inflammatory lacrimal gland mass in both lacrimal glands (**C**).

A 25-year-old female patient with a chief complaint of a palpable mass in both upper eyelids visited our medical center. The palpable mass and edema affected the temporal region of both upper eyelids and caused the patient considerable eye discomfort (Figure 1A). Visual acuity was 20/25 in the right eye and 20/30 in the left eye at the first visit. In the anterior segment examination, a protruding palpebral lobe of the lacrimal gland was noted in both eyes (Figure 1B). Fundus examination produced no specific findings. Contrast-enhanced orbital computed tomography (CT) revealed bilateral diffuse hypertrophy of the lacrimal glands (Figure 1C).

Upper eyelid swelling had not improved in either eye with steroid treatment; thus, we performed a lacrimal gland biopsy via the transconjunctival incision. The biopsy results showed chronic inflammation of the lacrimal gland. Immunohistochemical staining of CD3 and CD20 showed lymphocyte infiltration. Blood test results were positive for antinuclear antibodies; other rheumatoid factors, such as the anti-Ro/La antibody, were within normal limits.

**Figure 2:**
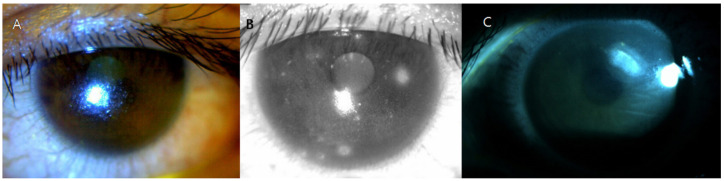
Postoperative slit lamp images of the cornea from Case 1. Severe dry eye disease had developed (**A**). Punctate corneal erosions were noted (**B**), and a corneal ulcer was evident at the postoperative follow-up (**C**).

At 3 weeks after the biopsy, the patient complained of extremely severe ocular pain and dry eye symptoms in both eyes. Under slit-lamp examination, the tear break-up time was reduced to <5 s in both eyes, and corneal erosions of grade 3 were found in the left eye (Figure 2A). To improve the symptoms, topical levofloxacin, 0.1% fluorometholone, 0.1% cyclosporine, and autologous serum eye drops were administered every 6 h, and topical carboxymethylcellulose sodium was administered every hour. Therapeutic contact lenses were placed in both eyes, and methylprednisolone was administered orally. After 5 months of treatment, the patient was readmitted with a corneal ulcer in the left eye (Figure 2B). At 9 months after the biopsy, the symptoms persisted; thus, permanent surgical punctal occlusion of both the upper and lower punctum of both eyelids was performed. Intractable DES and filament keratitis developed. The DES symptoms have yet to resolve for this patient as of the 2.5-year follow-up examination.

**Figure 3:**
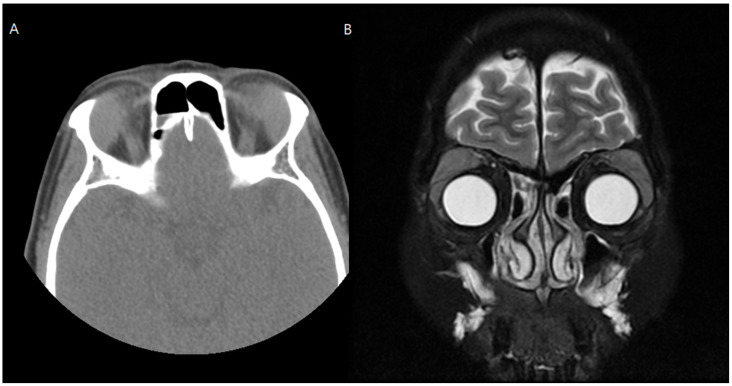
Preoperative computed tomography (**A**) and magnetic resonance imaging (**B**) of Case 2. Enlarged lacrimal glands were noted in both orbits.

A 52-year-old female patient with a chief complaint of swelling in both upper eyelids accompanied by diplopia visited our medical center. In orbital CT and magnetic resonance imaging scans, bilateral lacrimal gland hypertrophy was observed (Figure 3). Incisional biopsy of the bilateral lacrimal gland was performed to differentiate a diagnosis of lacrimal gland lymphoma from that of immunoglobulin G4 (IgG4)-related ophthalmic disease. The lacrimal gland tumor was approached via transconjunctival incision, and a biopsy was performed. Lacrimal gland biopsy with immunohistochemical staining of IgG4 showed a number of benign cells. Her blood test results showed increased IgG4 levels, and IgG4-related lacrimal adenitis was confirmed.

**Figure 4:**
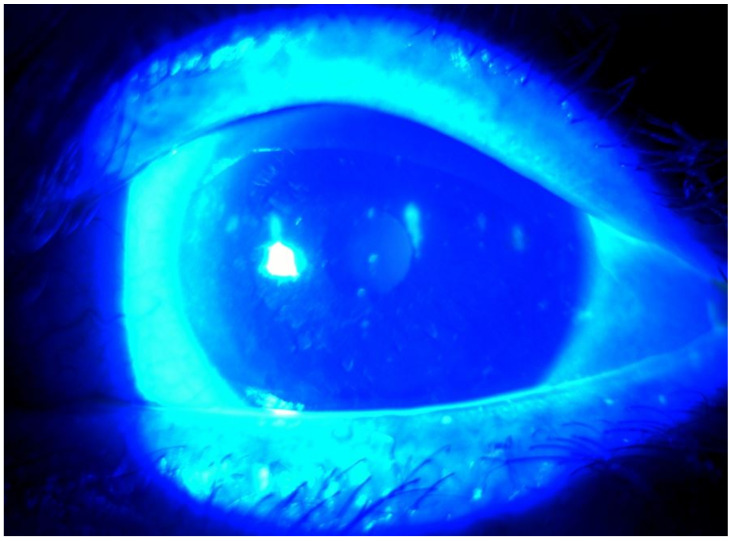
Postoperative slit lamp images of the cornea in Case 2. Severe dry eye disease of the left eye had developed postoperatively, and punctate corneal erosion was noted.

After the surgery, methylprednisolone was administered orally to control orbital inflammation. At 1 week after the surgery, severe DED occurred in the patient’s left eye (Figure 4). Therapeutic contact lenses were applied due to intractable ocular pain. On day 40 after the biopsy, the tear break-up time was reduced to 2–3 s in both eyes, and chemosis was observed in the temporal conjunctiva of the left eye. Topical levofloxacin and 0.1% fluorometholone, 0.1% cyclosporine, and autologous serum eye drops were applied in the left eye. At 6 months after the biopsy, corneal erosion of both eyes persisted, and findings of filamentary keratitis were observed in the left eye. Intense pulsed light (IPL) therapy was attempted at another clinic, as the conventional DED treatment for more than 6 months had been ineffective. However, there was no improvement in dry eye symptoms after the IPL treatment. Despite aggressive therapies to protect the ocular surface, the patient still suffers from aqueous-deficient DES. Currently, unaided visual acuity is 20/20 in the right eye and 20/22 in the left eye, and DES persists in both eyes, as of the follow-up in January 2018.

## 3. Discussion

In the present report, we describe two cases that suffered from refractory DES after transconjunctival partial excision of the palpebral lobe of the lacrimal gland. Despite all efforts to relieve ocular pain, the surgical intervention significantly impaired the quality of life of these patients. The first reason we report such cases is in the hopes that these cases will provide additional information regarding the main lacrimal gland’s role in aqueous-deficient DES. The second reason is that a review of such intractable cases and related literature may alert those less experienced in orbital surgery.

Unless the mass is confined to the palpebral lobe, the lacrimal gland should be biopsied from the orbital lobe, as this avoids damage to the secretory ductules and provides enough tissue for obtaining representative samples [8]. However, both patients in the present study wanted transconjunctival incision to minimize postoperative skin scars; further, slit-lamp examination showed a prominent enlarged palpebral lobe of the lacrimal gland. Thus, the biopsy could be performed easily with good visualization.

Maitchouk et al. [9] studied the effect of lacrimal gland removal on basal and reflex tear production in the squirrel monkey. Unilateral main lacrimal glands were removed from six squirrel monkeys, and tear production was observed through various methods. The results showed that after the lacrimal gland resection, secretion from the main lacrimal glands decreased; however, increased secretion from the accessory lacrimal glands prevented aggravation of DES. Conclusively, they showed that tears from the accessory lacrimal glands are sufficient to maintain a stable tear layer on the cornea [8]; thus, basal tear flow is made up of fluid from both the main and accessory lacrimal glands.

The patients in the present report presented no obvious reasons why tear production from the accessory lacrimal glands would have been impaired. Thus, we do not know why the patients suffered from intractable DES for such a long time after the surgery. Although a transconjunctival incision was made, the main lacrimal gland still remained. Sjögren’s syndrome was ruled out in both patients, and IgG4-related lacrimal adenitis did not impair the accessory lacrimal glands. DES is the most typical complication of the removal of the main lacrimal gland; however, this condition can be relieved over time in most cases. Previous experimental animal studies have shown that DES occurs temporarily after removal of main lacrimal gland and that the generation of tears normalizes as time goes by [9,10]. Previous clinical studies reporting that the main complication after partial palpebral dacryoadenectomy or total dacryoadenectomy was dry eye showed that all patients with symptomatic DES could be managed adequately with topical artificial tears [11,12].

Bhattacharya et al. [10] explored the tear compensation mechanism in rabbits with bilateral resection of the main lacrimal gland. They resected the main lacrimal glands in rabbits and performed ocular surface staining and tear secretion analysis, evaluating the expression of interleukin-1ß, tumor necrosis factor-α, matrix metalloproteinase-9, and aquaporin (AQP) 4 and AQP5 proteins in corneal and conjunctival epithelial cells over 4 months. The study results showed that after the occurrence of acute DES, the tear-secreting function recovered eventually [9]. The study also indicated the possible involvement of AQPs in compensatory tear fluid production through the upregulation of AQP4 and AQP5 expression in rabbit conjunctival epithelium.

Recently, Kim et al. [13] introduced a new method for direct observation of tear secretion from the palpebral lobes of the main lacrimal glands and Wolfring glands, one of the accessory lacrimal glands, using fluorescein dye. Interestingly, the mean number of excretory openings of the palpebral lobe and the mean tear flow rate from the palpebral lobe were significantly impaired in the dry eye group, compared to the healthy subject group. By contrast, the mean tear flow rate from the Wolfring glands did not differ between the two groups [13]. Jones [5] stated that the main lacrimal gland is responsible only for reflex tearing, whereas the accessory glands of Kraus and Wolfring provide basal tear secretion. In Kim and colleagues’ study [13], the tear flow rate from the Wolfring glands (0.07–0.09 µL/min) was very low compared to the normal tear flow rate (1.2 µL/min); thus, the authors suggested that tear secretion from only the accessory lacrimal gland is not sufficient. The cases in the present study support this hypothesis that tear secretion from the main lacrimal glands is more important.

Although the transconjunctival approach produces adequate biopsy specimens of the lacrimal gland in a majority of patients [14], a previous study showed that the incidence of DES was highest in the patients whose palpebral lobe was removed due to tumor involvement of lacrimal gland palpebral lobe [11]. Lacrimal gland inflammation is a condition that requires a biopsy for diagnosis [15]. In addition, incisional biopsy for inflammatory dacryoadenitis may be therapeutic [12]. Mombaerts et al. [12] described the surgical outcomes of patients with idiopathic dacryoadenitis, in whom surgical debulking of the lacrimal gland had been performed. The first reason why debulking improves inflammation in dacryoadenitis is that proinflammatory cytokines are produced early at the site of the surgical trauma to initiate the wound-healing process, which may help reduce the inflammation associated with dacryoadenitis [16]. Another possible mechanism is that surgically decreasing the volume of the inflammatory mass may help to improve the clinical signs and symptoms [12]. Mombaerts et al. [11] stated that debulking surgery did not lead to additional DES; however, the surgery should be performed such that the palpebral lobe is preserved via transcutaneous incision, as the excretory ducts pass through the palpebral lobe [12,17]. According to a recent report, lacrimal gland duct epithelia may play an important role in tear fluid production, because duct cells possess numerous secretory granules, as observed in acinar cells [18]. Furthermore, the inflammatory tumor disables lacrimal gland function by destroying acinic and tubular structures [19].

In general, for a transconjunctival approach to the lacrimal gland, a lacrimal gland biopsy is performed by incision of the superficial layer of the lacrimal gland starting with the conjunctival fornix. In 2005, Langsdon et al. [20] introduced a procedure to access the zygomatico-frontal limb of the fracture through a superior-lateral transconjunctival upper eyelid incision. The incision was made in a location superior to Whitnall’s tubercle and lateral to the muscular fibers of the levator palpebrae muscle. Guerra et al. [21,22] described that there is a conjunctiva covering a thin extension of the levator aponeurosis described as a “bare area” in the medial area of the upper lid. Langsdon et al. [20,23] suggested that an anatomical structure similar to this “bare area” was also present in the lateral upper lid. In their recent report [23], it was confirmed that the lacrimal gland was protected when the lateral orbital rim was approached through the lateral extension area of the levator aponeurosis, and there was no appearance of ptosis, lacrimal abnormalities, or dry eye syndrome after the operation.

In the present study, we report two cases of refractory DES after transconjunctival excision of the palpebral lobe of the lacrimal gland. The main lacrimal glands secrete a large fraction of the aqueous component of tears and are responsible for basal and reflex tearing. The extent of the contribution of accessory lacrimal glands to tear secretion at the ocular surface still remains unclear, and research regarding lacrimal gland function and pathology remains inadequate [24]. There is a limited understanding of the mechanism underlying lacrimal gland inflammation and damage, and case reports of DES complications after lacrimal gland surgery are similarly limited. We believe that this report provides additional information regarding the main role of the lacrimal gland in aqueous-deficient DES. Furthermore, all orbital surgeons should be aware of the possibility of such postoperative complications.

## Data Availability

Data and material are available on reasonable request.
